# High plasma level of S100A8/S100A9 and S100A12 at admission indicates a higher risk of death in septic shock patients

**DOI:** 10.1038/s41598-019-52184-8

**Published:** 2019-10-30

**Authors:** Christelle Dubois, Dominique Marcé, Valérie Faivre, Anne-Claire Lukaszewicz, Christophe Junot, François Fenaille, Stéphanie Simon, François Becher, Nathalie Morel, Didier Payen

**Affiliations:** 1 Service de Pharmacologie et Immunoanalyse (SPI), CEA, INRA, Université Paris-Saclay, Gif-sur Yvette, 91191 France; 2 Université Paris 7 Cité Sorbonne; UMR INSERM 1160, 110 Avenue de Verdun, Paris, 75010 France; 3 Department of Anesthesiology & Critical Care, Hôpital Lariboisière, Assistance publique-Hôpitaux de Paris (AP-HP), Paris, 75010 France

**Keywords:** Prognostic markers, ELISA

## Abstract

Biomarkers in sepsis for severity, prediction of outcome or reversibility of organ dysfunction are warranted. Measurements of plasma DAMP levels at admission can reflect the severity of cellular damage in septic shock, which might predict the prognosis and reduce the risk of overtreating patients with costly therapies. We measured plasma levels of two DAMPs, S100A8/S100A9 and S100A12 during the first 24 h of admission of septic shock patients. Forty-nine septic shock patients with a similar SOFA scores were selected from our sepsis database to compare a similar proportion of survivors and non-survivors. Plasma levels of S100A8/S100A9 and S100A12 were compared with healthy volunteers using in-house ELISA. Plasma levels of S100A8/S100A9 and S100A12 (5.71 [2.60–13.63] µg/mL and 0.48 [0.22–1.05] µg/mL) were higher in septic shock patients than in healthy volunteers (1.18 [0.74–1.93] µg/mL and 0.09 [0.02–0.39] µg/mL) (P < 0.0001 and P = 0.0030). Levels of S100A8/S100A9 and S100A12 in non-survivors at day 28 (11.70 [2.85–24.36] µg/mL and 0.62 [0.30–1.64] µg/mL) were significantly higher than in survivors (4.59 [2.16–7.47] µg/mL and 0.30 [0.20–0.49] µg/mL) (P = 0.0420 and P = 0.0248) and correlated well (Spearman r = 0.879, P < 0.0001). The high level of plasma calgranulins at admission in septic shock, were higher in non-survivors compared to survivors. These markers could indicate a higher risk of death when SOFA scores are similar and help the stratification of patients for improved care and therapy selection.

## Introduction

The definition of septic shock has recently evolved to allow precise selection of severely ill patients with organ failure related to suspected infection^1^. In 2016, in the framework of the Third International Consensus Conference, the Sepsis-3 group updated the definition of sepsis and promoted the use of the SOFA (Sequential Organ Failure Assessment) score^1^, which highlights inflammation, induced cell damage and organ dysfunction. The SOFA score is a morbidity severity score, but is not associated with substantial cell death^2^ and cannot predict the reversibility of the patient’s condition. It is crucial that biomarkers measured at admission in intensive care unit (ICU) may predict which patients will survive, thereby avoiding overuse of costly adjuvant treatments. Many arguments have been reported on the role of DAMPs (Damage-Associated Molecular Patterns) in cell dysfunction or damage^3^ to maintain systemic inflammation by interaction with PRRs (Pattern Recognition Receptors)^4^. In addition to systemic inflammatory markers as IL-6 (Interleukin-6) or IL-10 (Interleukin-10) frequently used to characterize inflammatory status, DAMPs may provide information on cell lesions uncorrelated with inflammatory markers^4^. Conversely, IL-6 and IL-10, which are frequently used to characterize inflammatory status, correlate poorly with cell dysfunction markers^5–8^. We hypothesized that the plasma calgranulins S100A8/S100A9 and S100A12 might be informative as DAMPs because: (i) their mRNA expression increases in septic shock^9,10^; (ii) the complex S100A8/S100A9 is related to myeloid cells whereas S100A12 is more restricted to granulocytes^11^; and (iii) the effect of complex S100A8/S100A9 is mediated via TLRs (Toll-Like Receptors) and RAGE (Receptor for Advanced Glycation End-product), whereas the mediation effect occurs purely via RAGE for S100A12^12^. In this context, the aims of this study were: 1- to produce monoclonal antibodies (mAbs) against S100A8, S100A9 and S100A12 for implementation of ELISA (Enzyme-Linked Immunosorbent Assay) to measure the S100A8/S100A9 complex and S100A12 protein; 2- to compare the plasma levels of these two calgranulins in septic shock patients versus age-matched controls; 3- to compare calgranulins levels during the first 24 h after admission for septic shock between survivors and non-survivors; 4- to evaluate the value of adding calgranulins levels to the initial SOFA score for outcome distribution.

## Results

### Assay development and validation

MAbs against recombinant S100A8, S100A9 and S100A12 proteins were produced and ELISAs were developed. The best sandwich immunoassays were selected according to the strongest signal to noise ratio observed with a commercial recombinant human S100A8/S100A9 heterodimer or S100A12 and plasma samples. Standard curves were established with the recombinant S100A8/S100A9 and S100A12 proteins in casein buffer (not shown). The limit of detection of the heterocomplex S100A8/S100A9-ELISA was determined as 98 pg/mL and the lower limit of quantification as 262 pg/mL. Intra-assay and inter-assay coefficients of variation for S100A8/S100A9 were 4.0% and 12.3%, respectively. The limit of detection for the S100A12-ELISA was determined as 2 pg/mL with a lower limit of quantification of 146 pg/mL. The intra-assay and inter-assay coefficients of variation for S100A12 were 2.4% and 14.0%, respectively.

### Patients and plasma calgranulin levels

**Figure 1:**
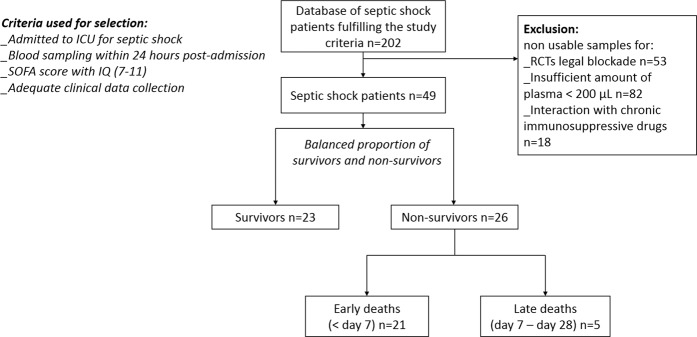
Schematic representation of septic shock patient’s selection from septic database.

**Figure 2:**
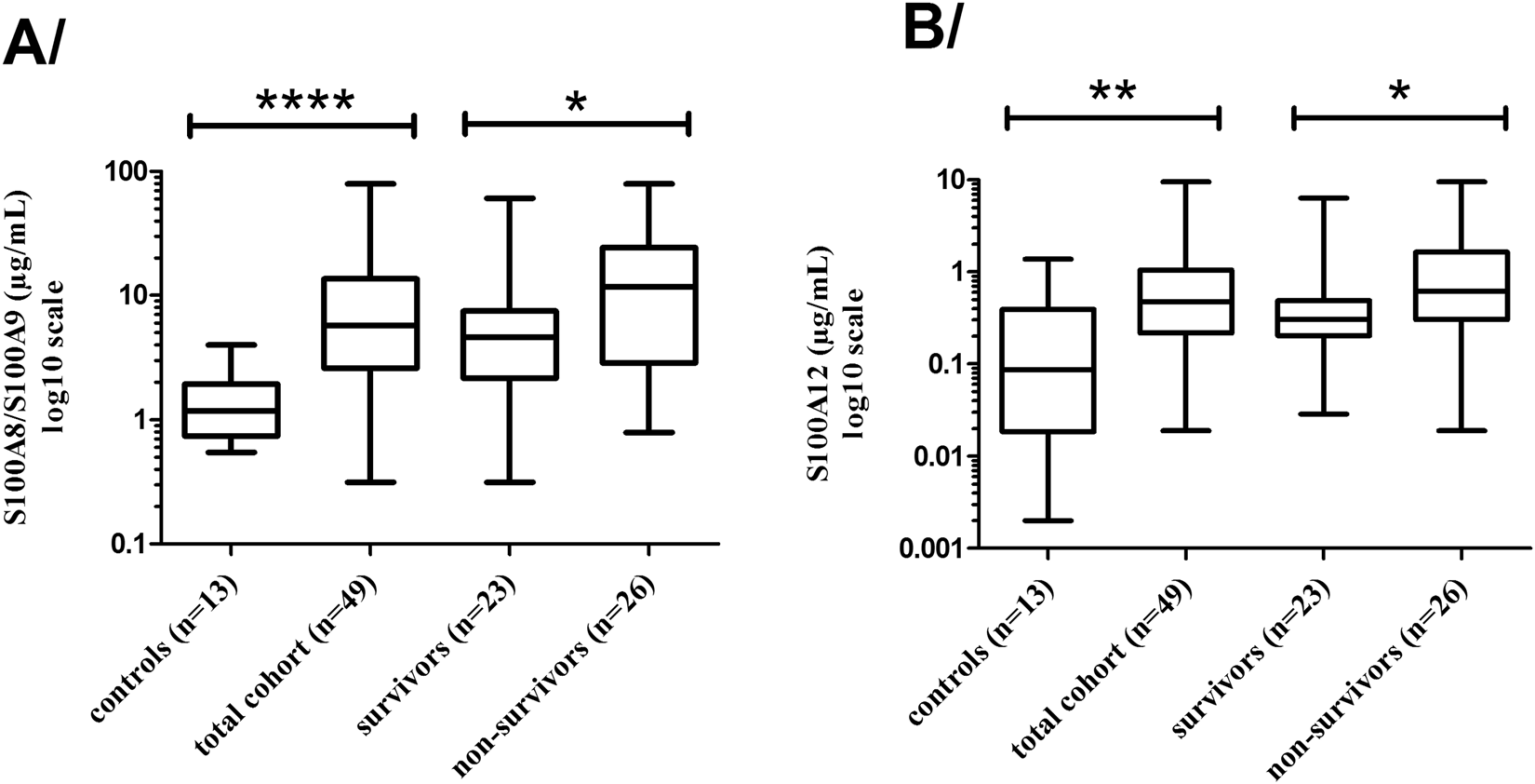
A/S100A8/S100A9 complex levels in controls, total cohort, survivors and non-survivors analyzed using our in-house S100A8/S100A9 ELISA. B/S100A12 protein levels in controls, survivors and non-survivors analyzed using the in-house S100A12 ELISA. Mann-Whitney test. Asteriks indicate P values: ****P < 0.0001, **0.001 < P < 0.01, *0.01 < P < 0.05.

As shown in Figure 1, the patients were selected from our clinical database and plasma bank on the following criteria: presence of septic shock at admission to ICU; blood samples withdrawn within the first 24 h post-admission for septic shock; informative clinical data; a SOFA score (excluding the neuro component) ranging from 7 to 11. From our previously published results^9^, we estimated that 49 patients with a balanced outcome (survivors and non-survivors) might be convenient to achieve the goal. In addition, among the non-survivors at day 28, we looked at the potential differences in calgranulins between early (before day 7) versus later (after day 7) deaths. The overall clinical characteristics of the selected patients were as follows: 100% were mechanically ventilated and 15% had a renal replacement therapy. Among the cohort, the proportion for a surgical/medical context were 70/30%, with a length of stay in ICU at 13.2 (8.2–17.6) days. Positives cultures were obtained in 62.5% of cases with 18.7% Gram+, 25% Gram−, 18.7% polymicrobial, and 37% with non-identified bacteria. Table 1 shows the clinical characteristics at admission of the selected patients who were similar in age, SOFA score, origin of infection and comorbidities (P-value > 0.05). The study was focused on septic shock patients because of the demonstrated highest level of death compared to patients with no shock^1^. Similar SOFA scores (9 [7–11]) as a selection criteria was chosen to limit the impact of this cofounding factor on outcome at day 28. As a consequence, calgranulins levels did not correlate with SOFA scores (R² = 0.031 for S100A8/S100A9 and R² = 0.0040 for S100A12) and were analyzed as DAMPs with similar severity (SOFA scores). The median values for both S100A8/S100A9 and S100A12 are reported in Table 1 and in Figure 2. Compared to age-matched controls, the whole cohort of patients had highly significant higher levels of both calgranulins (P < 0.0001 and P = 0.0030, respectively). The non-survivors at day 28 had significantly higher levels of S100A8/S100A9 (P = 0.0420) and S100A12 (P = 0.0248) than survivors at day 28 (Table 1, Figure 2).

**Table 1:** Patient characteristics, comorbidities and plasma calgranulin (S100A8/S100A9 & S100A12) levels 24 h post admission for the whole cohort, the survivors and non-survivors at day 28. NA: non-applicable. Data were expressed in median and interquartiles (IQ) or absolute numbers. (Mann-Whitney test).

Parameter		control n = 13	total cohort n = 49	survivors n = 23	non-survivors n = 26	P-value (controls *vs* cohort)	P-value (survivors *vs* non-survivors)
Age (y/o)		61 (43–74)	66 (54.5–78)	63 (23–80)	70.50 (56.75–77.25)	0.287	0.325
Sex M/F		12/13	27/49	13/23	14/26	0.025	0.777
SOFA		NA	9 (7–11)	9 (7–11)	10 (7–12)	NA	0.212
7-day mortality		0/13	21/49	0/23	21/26	NA	NA
28-day mortality		0/13	26/49	0/23	26/26	NA	NA
**Origin of infection**							
Abdominal sites		NA	35%	30%	38%	NA	0.568
Urinary		NA	6%	9%	4%	NA	0.500
Respiratory		NA	47%	48%	46%	NA	0.917
Others		NA	12%	13%	12%	NA	0.888
**Comorbidities**							
*Cardiovascular*	Hypertension	0%	33%	22%	42%	NA	0.132
	Coronary disease	0%	12%	9%	15%	NA	0.491
	Cardiac failure	0%	20%	13%	27%	NA	0.239
	Others	0%	2%	0%	4%	NA	0.368
*Diabetes*		0%	22%	17%	27%	NA	0.437
*Chronic pulmonary diseases*		0%	10%	17%	4%	NA	0.126
*Neurology diseases*	Stroke	0%	2%	4%	0%	NA	0.306
	Intracerebral bleeding	0%	2%	4%	0%	NA	0.306
	Others	0%	10%	17%	4%	NA	0.126
*Gastroenterology diseases*		0%	12%	9%	15%	NA	0.314
Cancers		0%	14%	22%	8%	NA	0.170
*Chronic viral infection* (HIV, VHB,C)		0%	4%	9%	0%	NA	0.136
**Calgranulins concentrations**							
S100A8/S100A9 (µg/mL) 1^st^ 24 h		1.18 (0.74–1.93)	5.71 (2.60–13.63)	4.59 (2.16–7.47)	11.70 (2.85–24.36)	<0.0001	0.042
S100A12 (µg/mL) 1^st^ 24 h		0.09 (0.02–0.39)	0.48 (0.22–1.05)	0.30 (0.23–0.49)	0.62 (0.30–1.64)	0.0030	0.024

Table 1 shows the delay between the date of death and admission. The large majority of deaths occurred before day 7 (21/26) suggesting different mechanisms for late deaths (>day 7)^4^. Table 2 compares the median SOFA score and calgranulin levels in survivors and in patients who died earlier and later. At a similar median SOFA score, the survivors had a significantly lower level of both calgranulins than early and day 28 non-survivors.

**Table 2:** Comparison of the median values for S100A8/S100A9 and S100A12 and SOFA score in survivors, non-survivors before day 7 and non-survivors at day 28. Data were expressed in median and interquartiles (IQ) or absolute numbers. (Mann-Whitney test).

Parameter	survivors (n = 23) A	non-survivors B ( < day 7) (n = 21)	late non-survivors (n = 5) C	P-value (A vs B)	P-value (A vs C)	P-value (B vs C)
SOFA	9 (7–11)	11 (7–12)	7 (6–10)	0.053	0.605	0.166
S100A8/S100A9 (µg/mL) 1^st^ 24 h	4.59 (2.16–7.47)	13.02 (5.13–37.56)	3.03 (1.10–15.36)	0.013	0.857	0.152
S100A12 (µg/mL) 1^st^ 24 h	0.30 (0.20–0.49)	0.63 (0.41–2.12)	0.33 (0.07–1.63)	0.001	0.473	0.435

**Figure 3:**
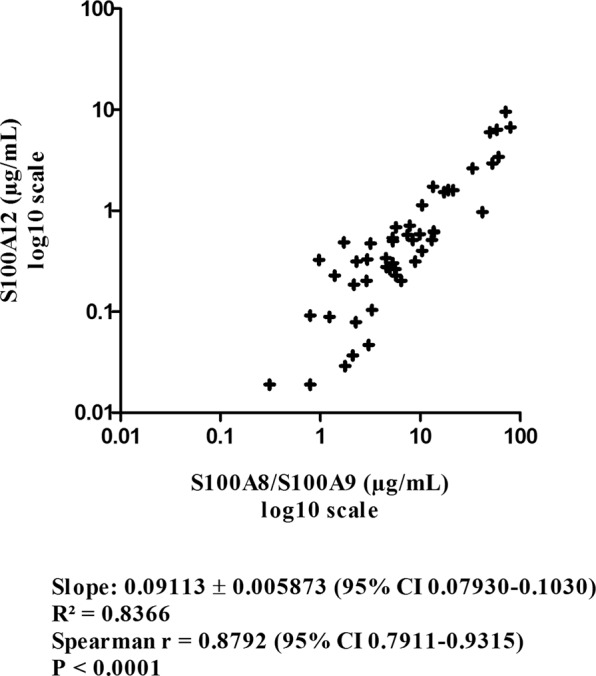
Linear correlation between plasma levels of S100A8/S100A9 and S100A12 on a log_10_ scale (R² = 0.8366, P < 0.0001 and Spearman r = 0.8792, P < 0.0001).

Figure 3 shows the strong linear correlation (R² = 0.8366; P < 0.0001) between plasma levels of S100A8/S100A9 and S100A12, suggesting a simultaneous release of these molecules potentially mediated by similar signalization mechanisms in septic shock despite their different cell origins.

**Figure 4:**
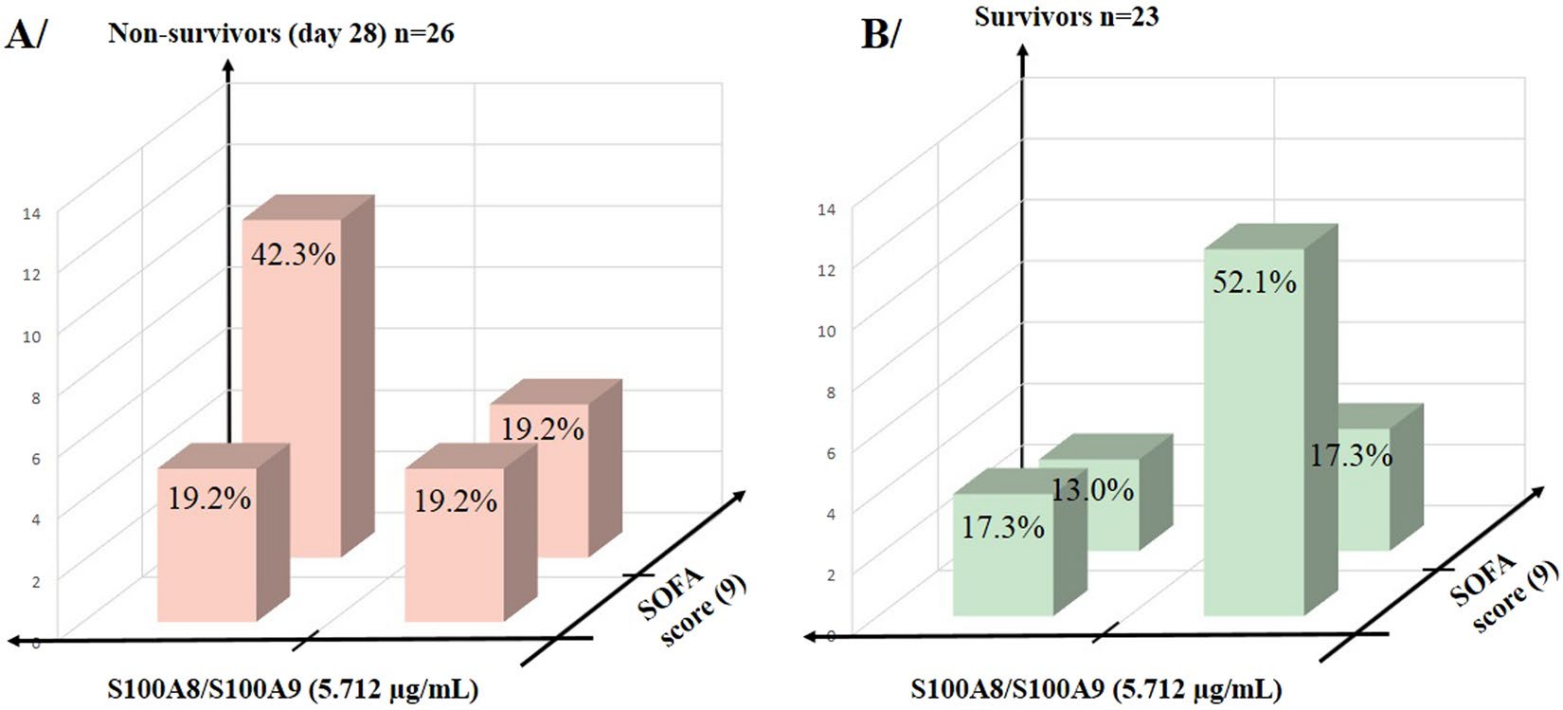
Three-dimensional representation of the patient distribution according to the median values of plasma S100A8/S100A9 levels (5.712 µg/mL) and the SOFA score (9) measured, leading to 4 groups in survivors and non-survivors. The percentage represents the fraction of events (non-survivors, survivors) referring to the total number of patients in each group. A/Distribution of non-survivors at day 28 (n = 26). B/Distribution of survivors at day 28 (n = 23).

Figure 4 displays the distribution of non-surviving patients at day 28 (Fig. 4A) and of survivors (Fig. 4B) as a function of median SOFA scores and S100A8/S100A9 (S100A12 in Supplementary Fig. S1). The three-dimensional combination of SOFA score and calgranulin levels improved the stratification of septic shock patients into 4 groups: (i) SOFA score and calgranulin level higher than the median, (ii) SOFA score and calgranulin level lower than the median, (iii) higher SOFA score and lower calgranulin, and (iv) lower SOFA score and higher calgranulin. The distribution of death differed significantly within the 4 groups (probability for mortality log-rank test: χ^2^ = 5.132, df = 1, P = 0.0235). At both high SOFA score and S100A8/S100A9 level (>median values), the proportion of global death (and early deaths Supplementary Fig. S2) was elevated (42.3%). Conversely, the proportion of survivors fell to 13.0%. At both low SOFA score and S100A8/S100A9 level (<median values), the proportion of death was 19.2% (9.5% for early deaths; Supplementary data Fig. S2) with a large increase of proportions of survivors (52.1%). Similar results were obtained for S100A12 (Supplementary Fig. S1). Addition of high calgranulin levels to high SOFA scores may improve the prediction of deaths at day 28 and at day 7 in septic shock patients, suggesting a higher risk of death when calgranulins are released at the early phase.

## Discussion

The choice of biomarkers in septic shock patients remains difficult, due to the complexity of the pathophysiology, the fast disease progression and the difficulty of selecting the main targets. The biomarkers may provide information on the intensity of systemic inflammation, organ failure risk, outcome, and also on tissue or cell damage. The latter seems to be more related to the concept of DAMPs released during cellular stress^3^. The level of such DAMPs may then differ from those observed for markers of systemic inflammation^4^. Among the DAMPs, we decided to measure the plasma level of two different calgranulins, which have been previously reported in septic shock^9,13–15^. Among the 20 members of the calgranulin protein family, S100A8/S100A9 and S100A12 were shown to be specifically linked to innate immune function^12^. S100A8 and S100A9 are released from granulocytes, monocytes and macrophages in the early differentiation stages^16^. By contrast, S100A12 appears more restricted to granulocytes^11^. The respective cellular effects of these two molecules are mediated by TLRs and RAGE receptors for the complex S100A8/S100A9 and exclusively by RAGE receptors for S100A12^12^. This justifies the measurement of the two protein plasma levels, which may activate endothelial and tissue cells differently according to expression of the receptor^4^.

Plasma levels of both S100A8/S100A9 and S100A12 are higher in septic shock patients than in age-matched controls^9,13,14^. The levels reached in the present study fit well with those reported for S100A8/S100A9^9^ in patients with septic shock and for S100A12^13^ in a cohort of septic patients (69% of patients with septic shock). When survivors were compared to non-survivors at a similar high SOFA score, the levels at the first day of admission were higher (>two-fold increase) in non-survivors for both molecules, despite their different sources and receptor targets^11,16^. Since the plasma protein level at the initial phase of septic shock did not correlate with S100A8 and S100A9 gene expression on peripheral white blood cells^9,16^, the observed higher levels may result from greater cellular damage related to a more intense cellular stress. The parallel release of these DAMP molecules may have different cellular consequences according to receptor availability and the different mechanisms of action. Figure 4 illustrates the combination between SOFA score and early S100A8/S100A9 levels together with mortality and survival rates (similar for S100A12 in Supplementary Fig. S1 online). This suggests that tissue damage has an additional impact on outcome compared to the intensity of inflammation, adding a mortality risk factor in septic shock patients. This may result from greater cellular damage induced by these molecules, some damage becoming irreversible. More investigations in larger cohorts of septic shock patients are needed to test this hypothesis. Thorough evaluation of cases of patients who died despite having plasma calgranulin levels below the median failed to find specific clinical conditions to explain the death.

Our study has some limitations. The most important relates to the small size of the study cohort, which precludes definitive conclusions before validation in a larger continuous prospective population of septic shock patients. Although the validation of biomarkers was not the goal of the study, the observed results point to the value of testing S100A8/S100A9 and S100A12 in a large population of septic shock patients. The second limitation concerns the method of patient selection applied to our database. Patients were selected on a priori criteria from a database to obtain a balanced rate of non-survivors *vs* survivors at day 28 at similar SOFA. This pilot study gave plasma values of S100A8/S100A9 and S100A12 associated with surviving and non-surviving patients that can then be used to design randomized clinical trials mainly focused on patients with a high risk of death.

## Conclusion

Measurements of two DAMP members of the calgranulin family using fully validated in-house ELISAs gave plasma level ranges of both calgranulins consistent with previous reports. The levels of these two molecules were strongly correlated, suggesting broad stimulation of both RAGE and TLR4 receptors. The elevation of these DAMPs was always associated with a higher risk of death than was the SOFA score alone. This suggests that measurement of S100A8/S100A9 and S100A12 levels at the early phase of septic shock could improve evaluation of tissue damage and indicate a higher risk of death, when added to the SOFA score.

## Methods

### Ethics statement

All experiments were performed in compliance with French and European regulations on the care of laboratory animals (European Community Directive 86/609, French Law 2001–486, 6 June 2001) and with the agreements of the Ethics Committee of the Commissariat à l’Energie Atomique (CEtEA ‘Comité d’Ethique en Expérimentation Animale’ No. 44) No. 15-046 delivered by the French Veterinary Services.

### Patients

The Ethics Committee of the Société de Réanimation de Langue Française (# CE SRLF 11 369) authorized for this study the use of an anonymized list of septic shock patients chosen in our database and informed consent was obtained from all participants and/or their legal guardians. The experiments were conducted in accordance with the SRLF Ethics committee guidelines and regulations. The cohort selection of 49 patients was made using the following criteria: (i) written informed consent from the patient or their legally authorized surrogate to be used anonymously for research purposes; (ii) at least 1 organ failure in addition to the shock (*i.e*. 2 organ failures) to match the Sepsis-3 definition^1^; (iii) frozen at −80 °C and well-traceable plasma withdrawn in the first 24 h post-admission in ICU. Severe infection was diagnosed by microbiological tests and/or on clinical grounds. Patients younger than 18 years old and those with active cancer or a hematologic malignancy were excluded. We selected a balanced proportion of survivor and non-survivor plasma samples with a high SOFA score (exclusion of neuro-component) (9 [7–11]). As a consequence, the proportion of non-survivors (n = 26) *vs* survivors (n = 23) at day 28 was the result of our selection and did not reflect the real death rate of septic shock patients. We compared the results obtained in our cohort with those for an age-matched group of healthy volunteers (n = 13) as a control group, given the influence of age on the plasma levels of calgranulins^17^.

### ELISA of S100A8/S100A9 and S100A12

Plasma levels of S100A8/S100A9 and S100A12 were measured by ELISA established with in-house mouse mAbs directed against recombinant human S100A8, S100A9 or S100A12 proteins expressed in *Escherichia coli*. Briefly, high-binding 96-well microplates (MaxiSorp™, Nunc) were coated overnight at 20 °C with 100 µL of a 5 µg/mL solution of anti-S100A8 mAb (clone A8–3) or anti-S100A12 mAb (clone A12-32) diluted in 50 mM phosphate buffer (pH 7.4). The plates were then saturated with casein buffer and stored at 4 °C until use. The wells were washed 5 times with 50 mM potassium phosphate/0.02% Tween-20 before addition of 100 µL of human plasma or recombinant S100A8/S100A9 or S100A12 proteins diluted in casein buffer. After 1-h incubation at room temperature, the plates were washed 5 times and 100 µL of biotinylated anti-S100A9 mAb (clone A9-111) or anti-S100A12 mAb (clone A12-37) at 100 ng/mL was added. After 1 h, the wells were washed and 100 µL of streptavidin poly-HRP was added to the wells for 30 min. The wells were washed 5 times and 100 µL of Ultra TMB-ELISA Substrate Solution was added. The reaction was stopped after 30 min by addition of 100 µL of 1 M H_2_SO_4_ and the absorbance was measured at 450 nm against a reference wavelength of 620 nm.

### Assay validations

The intra-assay coefficient of variation was determined by assaying six times on the same day a pooled plasma sample from the cohort of septic shock patients. The inter-assay coefficient was determined by repeating this experiment on three different days. The limit of detection was calculated as the concentration of recombinant S100A8/S100A9 heterodimer (R&D Systems, Minneapolis, MN, USA) or in-house S100A12 protein corresponding to the mean of 6 measurements of the casein buffer +3 standard deviations (99.9% confidence) and was determined from the standard curve fit. The limit of quantification was calculated as the concentration of recombinant protein corresponding to the mean of 6 measurements of the casein buffer +10 standard deviations (99.9% confidence).

### Statistical analysis

All data were analyzed using GraphPad Prism software (5.04). The data were expressed as median values with interquartile range (25–75) or percentage. Because of the large ranges of plasma concentrations and for better visualization, the results were expressed in log_10_ of the µg/mL concentration. Intergroup comparison was made using the non-parametric Mann-Whitney test. From the median values, the S100A8/S100A9 and S100A12 and elevated SOFA scores were stratified in high and low levels, which distributed the cohort of septic shock patients in 4 groups. A log-rank (Mantel-Cox) test was used to analyze the outcome difference within the 4 groups. A two-sided value of P < 0.05 was considered as statistically significant.

## Supplementary information

Figure S1 and Figure S2
