# Supplementary material for: High plasma level of S100A8/S100A9 and S100A12 at admission indicates a higher risk of death in septic shock patients
Source: Sci Rep. 2019 Oct 30;9:15660. doi: 10.1038/s41598-019-52184-8 (PMC6821805; doi:10.1038/s41598-019-52184-8)
Supplement: Supplementary file 1 — Figure S1 and Figure S2 [file 41598_2019_52184_MOESM1_ESM.docx]

**Supplementary data**

**High plasma level of S100A8/S100A9 and S100A12 at admission indicates a higher risk of death in septic shock patients**

**Christelle Dubois^1^, Dominique Marcé^1^, Valérie Faivre^2^, Anne-Claire Lukaszewicz^2^, Christophe Junot^1^, François Fenaille^1^, Stéphanie Simon^1^, François Becher^1^, Nathalie Morel^1^*, Didier Payen^2^***


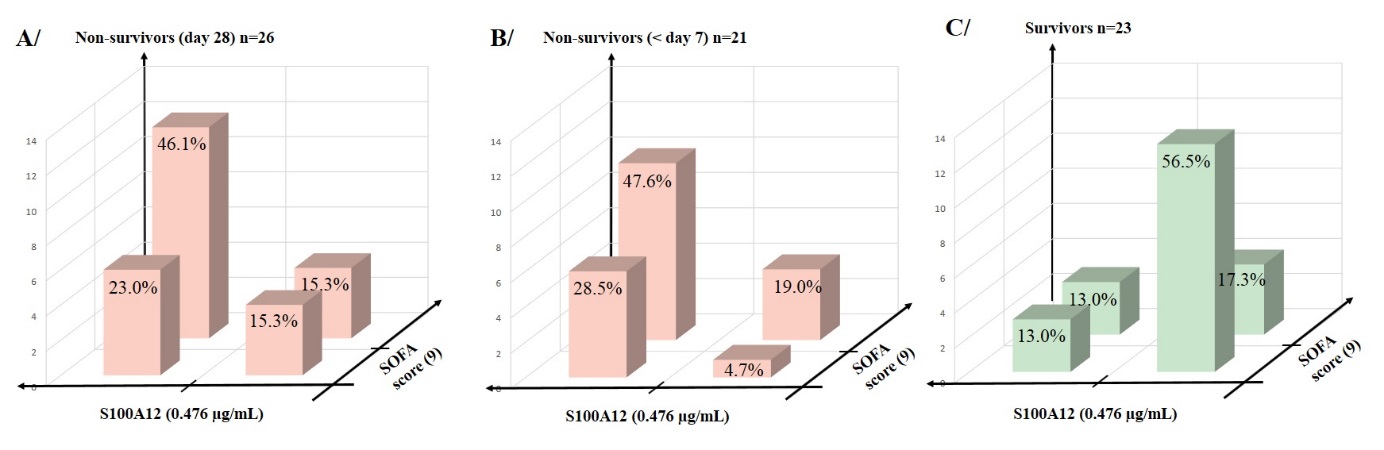


**Supplementary Figure S1**

Schematic tridimensional representation of the patients’ distribution according to the median values of S100A12 (0.476 µg/mL) plasma levels and SOFA score (9) leading to 4 groups. The % represents the fraction of events (non-survivors, survivors) referring to the total number of patients in each group. The probability of death differed significantly within the 4 groups (probability for mortality log-rank test: χ^2^ = 7.394, df = 1, P = 0.0065)

A/ Distribution of non-survivors at day 28 (n = 26).

B/ Distribution of non-survivors dying within the first 7 days (n = 21).

C/ Distribution of survivors at day 28 (n = 23).

**
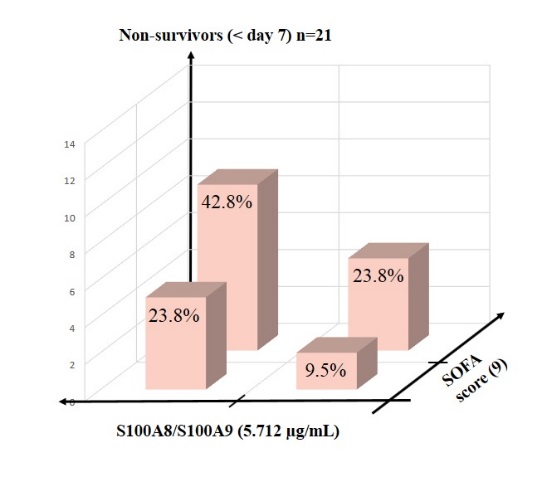
**

**Supplementary Figure S2**

Schematic tridimensional representation of the non-survivors dying within the first 7 days (n=21) distribution according to the median values of S100AA8 (5.712 µg/mL) plasma levels and SOFA score (9) leading to 4 groups. The % represents the fraction of events (non-survivors) referring to the total number of early deaths.
